# Genome-Wide Identification and Expression Profile of the HD-Zip Transcription Factor Family Associated with Seed Germination and Abiotic Stress Response in *Miscanthus sinensis*

**DOI:** 10.3390/genes13122256

**Published:** 2022-11-30

**Authors:** Jiayi Chen, Qifan Ran, Zhongfu Yang, Ying Zhou, Zhihang Yuan, Huiqi Lai, Jing Wang, Gang Nie, Yongqun Zhu

**Affiliations:** 1 Department of Forage Breeding and Cultivation, College of Grassland Science and Technology, Sichuan Agricultural University, Chengdu 611130, China; 2 Institute of Grassland Sciences, Chongqing Academy of Animal Science, Chongqing 402460, China; 3 Institute of Agricultural Resources and Environment, Sichuan Academy of Agricultural Sciences, Chengdu 610066, China

**Keywords:** *Miscanthus sinensis*, HD-Zip transcription factor, abiotic stress, seed germination, gene expression

## Abstract

*Miscanthus sinensis* is an ornamental grass, non-food bioenergy crop, and forage with high feeding value. It can adapt to many kinds of soil conditions due to its high level of resistance to various abiotic stresses. However, a low level of seed germination restricts the utilization and application of *M. sinensis*. It is reported that the Homeodomain-leucine zipper (HD-Zip) gene family participates in plant growth and development and ability to cope with outside environment stresses, which may potentially regulate seed germination and stress resistance in *M. sinensis*. In this study, a complete overview of *M. sinensis HD-Zip* genes was conducted, including gene structure, conserved motifs, chromosomal distribution, and gene duplication patterns. A total of 169 members were identified, and the HD-Zip proteins were divided into four subgroups. Inter-chromosomal evolutionary analysis revealed that four pairs of tandem duplicate genes and 72 segmental duplications were detected, suggesting the possible role of gene replication events in the amplification of the *M. sinensis HD-Zip* gene family. There was an uneven distribution of *HD-Zip* genes on 19 chromosomes of *M. sinensis*. Also, evolutionary analysis showed that *M. sinensis HD-Zip* gene family members had more collinearity with monocotyledons and less with dicotyledons. The gene structure analysis showed that there were 93.5% of proteins with motif 1 and motif 4, while motif 10 was only found in group IV. Based on the cis-elements analysis, it appeared that most of the genes were related to plant growth and development, various hormones, and abiotic stress. Furthermore, qRT-PCR analysis showed that *Misin06G303300.1* was significantly expressed in seed germination and *Misin05G030000.1* and *Misin06G303300.1* were highly expressed under chromium, salt, and drought stress. Results in this study will provide a basis for further exploring the potential role of *HD-Zip* genes in stress responses and genetic improvement of *M. sinensis* seed germination.

## 1. Introduction

As a perennial rhizomatous grass of the C4 family, *Miscanthus sinensis* is cultivated as a forage, ornamental, and energy crop due to its high biomass, photosynthesis potential, and water use efficiency [1,2,3,4]. It is reported that *M. sinensis* can adapt to multiple kinds of soil conditions due to its strong resistance to various abiotic stresses, such as heavy metal, salt, and drought stress [5,6,7]. However, it is widely believed that seed dormancy hindered free germination, which widely existed in *M. sinensis* [8,9,10], and seed germination characteristics contribute to the establishment of *M. sinensis* [11,12]. The seeds of *M. sinensis* are small, heterogeneous [13,14], and under natural conditions, the germination rate of seeds is only about 65% [12]. Therefore, it is imperative to explore the genes related to *M. sinensis* seed germination for further genetic improvement. There is increasing evidence indicating that Homeodomain-leucine zipper (HD-Zip) proteins have played a critical role in plant growth and development [15,16]. The *HD-Zip* gene family is a transcription factor family only found in plants, and it comprises two conserved functional domains: a homologous domain (HD) that specifically binds to DNA, and a leucine zipper (LZ) motif mediating the formation of protein dimers [17]. Additionally, HD-Zip proteins can be divided into four subfamilies: HD-Zip I (two domains, including a highly conserved HD and a less conserved LZ), HD-Zip II (HD domains, LZ domains, CPSCEs, and N-terminal consensus sequences exhibit high conservation), HD-Zip III (all members share the conserved domain “MEKHLA”, conservative amino acids account for more than half, and a START domain and SAD domain are contained), and HD-Zip IV (in comparison to HD-Zip III, the LZ domain has a loop in the middle and MEKHLA has disappeared) [18].

In recent years, plants from a wide range of species have been analyzed and identified to the function with *HD-Zip* genes. In *Arabidopsis thaliana*, *ATHB2* acted as a light-induced factor, which negatively regulated and maintained the expression of transcription factors to prevent seed germination, as a gene of HD-Zip class I [19]. Furthermore, several HD-Zip proteins are involved in regulating the expression of *NCED9*, which can mediate the synthesis of ABA and gibberellin-dependent seed germination [20]. With the development of cotton roots, HD-Zip class I gene *GhHB1* transcripts accumulated at the early stage and decreased dramatically as they developed [21]. As an *Arabidopsis thaliana HD-Zip* subgroup I gene, *ATHB23* was controlled by GA and other activators such as *PHABULOSA* and participated in the establishment of polarity during leaf development [22]. As an HD-Zip IV gene in transgenic maize, the ectopic expression of *OCL1* led to polyphenotypic aberrations, among which the most significant phenotypic aberrations were significantly delayed flowering [23]. *VvHB58*, a gene of the transgenic Grape HD-Zip I subfamily, was involved in regulating fruit size and seed yield [24]. *ZeHB10*, *ZeHB11,* and *ZeHB12*, three genes belonging to the HD-Zip III family in Zinnia, were reported to participate in the synthesis of lignin and the formation of lateral tissues [25].

Furthermore, *HD-Zip* genes also participated in regulating stress-responsive mechanisms under abiotic conditions. In rice, *Oshox22* positively regulated salt and drought tolerance via ABA-dependent signal pathways as an HD-Zip I gene [26]. In *Arabidopsis thaliana*, *ATHB17* knockout lines showed ABA-insensitivity and drought-sensitivity, indicating that *ATHB17* responded to ABA and water-stress as an HD-Zip IV gene [27]. Moreover, TDF #170-1 and 170-1k, two proteins of the HD-Zip I subfamily in *Citrus sinensis* root, were significantly upregulated under manganese (Mn) toxicity, showing that they participated in Mn stress tolerance [28]. *SlHZ24* was a gene belonging to the tomato HD-Zip class I, and it was reported that the overexpression of *SlHZ24* could promote acetylsalicylic acid biosynthesis and resulted in enhanced oxidative stress tolerance [29]. Furthermore, Transgenic Arabidopsis improved osmotic tolerance when supplemented with *GaHDG11*, a gene of cotton HD-Zip class IV [30]. The silence of *OsHB4,* an *Oryza sativa* HD-Zip I transcription factor, was reported to reduce Cd accumulation in leaves and grains [31].

There is no research conducted on the *HD-Zip* gene family in *M. sinensis* despite the fact that *HD-Zip* genes have been proven to play a significant role in plant growth and development and abiotic stress. In this study, genome-wide identification, duplication, gene structure, and phylogenetic characteristics of *M. sinensis HD-Zip* genes were comprehensively analyzed based on the genome sequence of *M. sinensis.* In addition, the expression level of *HD-Zip* genes in seed germination and their responses to various abiotic stresses were also investigated. The results will be helpful to investigate the molecular mechanisms and the functional characteristics of *M. sinensis HD-Zip* genes associated with seed germination and abiotic stress.

## 2. Materials and Methods

### 2.1. Identification of HD-Zip Genes in M. Sinensis

The *M. sinensis* genome resource was downloaded from the PHYTOZOME database (https://phytozome-next.jgi.doe.gov/info/Msinensis_v7_1, accessed on 1 June 2022) [32]. To identify the *M. sinensis* HD-Zip members, the leucine zipper (LZ) domain (PF02183) and the Hidden Markov Model (HMM) file of the conserved HD domain of homeobox (PF00046) were obtained from the Pfam database (http://pfam.sanger.ac.uk/, accessed on 1 June 2022). Firstly, HMMER 3.0 was used to conduct a local BLSATP search against the proteome sequences of *M. sinensis* using the HMM profile as a query. An e-value cut-off of 1×E at minus 5 was used for the query. After removing all redundant sequences, all candidate protein sequences were confirmed by SMART (http://smart.emblheidelberg.de/smart/save_user_preferences.pl, accessed on 2 June 2022) tools and NCBI CDD (https://www.ncbi.nlm.nih.gov/Structure/bwrpsb/bwrpsb.cgi, accessed on 2 June 2022) for further verification.

Further, the CD length, molecular weight (MW), and theoretical isoelectric points (pI) of HD-Zip proteins were analyzed by ProtParam (https://web.expasy.org/protparam/, accessed on 2 June 2022), and the subcellular localization of the HD-Zip proteins was predicted by the online ProtComp 9.0 (http://linux1.softberry.com/berry.phtml, accessed on 3 June 2022) [33]. In order to predict the membrane-bound HD-Zip proteins, the TMHMM server v.2.0 (http://www.cbs.dtu.dk/services/TMHMM/, accessed on 3 June 2022) was used [34]. An analysis of the cis-acting regulatory elements was performed using PlantCARE (http://bioinformatics.psb.ugent.be/webtools/plantcare/html/, accessed on 3 June 2022) [35].

### 2.2. Phylogenetic Analysis and Classification of HD-Zip Genes

The HD-Zip protein sequences of *Arabidopsis thaliana* were obtained from the TAIR 11 [36] (https://www.arabidopsis.org/, accessed on 4 June 2022). Amino acid sequences of *M. sinensis* HD-Zip proteins at full-length were used for multiple alignments conducted by muscle and automatedly aligned in Trimal. The phylogenetic tree was constructed by IQ-TREE [37]. Model_finder was used to find the best tree composition model for VT + F + R7 [38], and ultrafast bootstrap was performed using 1000 replicates with the partial deletion model for gaps/missing data [39]. Phylogenetic analysis of the transcription factors of *M. sinensis* and *A. thaliana* shows that the *HD-Zip* genes in *M. sinensis* can be further subdivided into different subfamilies.

### 2.3. Gene Structure and Motif Composition of HD-Zip Genes

Through the online Gene Structure Display Server 2.0 tools, the exon-intron structures of *HD-Zip* genes were displayed graphically to gain insight into their structures. In order to identify the conserved motif in HD-Zip proteins of *M. sinensis*, the program Multiple Em for Motif Elicitation (MEME) was used with default settings [40].

### 2.4. Chromosomal Distribution, Gene Duplication, and Synteny Analysis of HD-Zip Genes

The chromosomal distribution information of the *HD-Zip* genes was obtained from the *M. sinensis* genome annotation. Additionally, MG2C was used to draw the chromosomal positions map of the *HD-Zip* genes and specific nomenclatures were assigned based on the order of chromosomes. An analysis of segmental and tandem duplications was carried out by a Multiple collinear scanning toolkits (MCScanX) with default parameters [41]. TBtools platform was used to create the corresponding plot using Dual Synteny Plot for MCscanx [42]. To discuss the synteny relationship of orthologous *HD-Zip* genes between *M. sinensis* and ten other plants (*Arabidopsis thaliana*, *Medicago truncatula*, *Glycine max*, *Trifolium repens*, *Sorghum bicolor*, *Bracypodium distachyon*, *Panicum hallii*, *Saccharun spontaneum*, *Zea mays,* and *Setaria italica*), the synteny analysis maps were generated with TBtools Dual Synteny Plotter (https://github.com/CJ-Chen/TBtools, accessed on 4 June 2022).

### 2.5. Plant Growth and Treatments

To detect the expression of *M. sinensis HD-Zip* genes during seed germination, mature seeds of *M. sinensis* ‘M20100819’ were placed into Petri dishes and cultured in incubators (day/night temperature of 30/20 °C, light and dark cycle of 16/8 h) [8,10]. Distilled water was poured every 24 h. Whole plant samples were taken at 0 h, 2 h, 1 d, 3 d, and 5 d respectively, three biological replicates were taken at each time point.

Furthermore, the expression levels of *HD-Zip* genes in *M. sinensis* root under stress conditions were determined. The seedlings were cultured in plastic pots with a matrix of quartz sand and watered with Hoagland’s nutrient solution every day. The growth conditions of plant materials were 28/25 °C, with a light and dark cycle of 12/12 h, respectively, in the growth chamber [43]. Three different stress treatments were carried out for six days when plants had 5–6 leaves, including salt stress (300 mmol/L NaCl), drought stress (20% PEG), and chromium stress (200 mg/L K_2_Cr_2_O_7_). Three biological repetitions for each treatment were used to collect root samples on days 0, 1, 3, and 6. As soon as the samples were collected, they were frozen in liquid nitrogen and then stored at −80 °C.

### 2.6. RNA Extraction, cDNA Synthesis, and qRT-PCR Gene Expression Analysis

In order to perform qRT-PCR analysis, total RNA was extracted with the help of an RNA extraction kit (Zymo Research, Beijing, China). Elimination of genomic DNA contamination was performed by DNase I (Zymo Research, Beijing, China). The concentration of RNA was determined with a Nanodrop 2000 spectrophotometer (Thermo Scientific, Waltham, MA, USA) and the integrity of RNA was monitored with agarose gel electrophoresis. With the 5X All-In-One RT MasterMix (Abm, Richmond, VA, USA), the first-strand cDNA was synthesized according to the manufacturer’s instructions.

The qRT-PCR primers (Table S1) were designed by Primer 3.0 software. The expression analysis of the selected genes was conducted by qRT-PCR with SYBR Premix ExTaq II (TaKaRa). *Unigene33024* was used as the reference gene in this qRT–PCR experiments [43]. The data were calculated by the 2^−(∆∆Ct)^ method [44].

## 3. Results

### 3.1. Identification of HD-Zip Genes in M. sinensis

A BLASTP search was conducted against the *M. sinensis* genome database with the HMM profile being used as a query to retrieve consensus protein sequences, and a total of 169 *M. sinensis HD-Zip* genes were identified. As can be found from Table S2, among these 169 HD-Zip proteins, the smallest protein was Misin05G360200.1, which contains 96 amino acids, and the largest was Misin17G240000.1 (which contains 1856 amino acids). Also, *HD-Zip* genes ranged from 288 bp (*Misin05G360200.1*) to 5568 bp (*Misin17G240000.1*) in length. The pI ranged from 4.38 (*Misin03G157300.1*) to 10.74 (*Misin15G189700.1*), with an average of 6.95. In addition, subcellular localization predicted that most of the *M. sinensis* HD-Zip proteins (~99.41%) were in the nucleus, and all proteins except protein Misin16G051700.1 were transmembrane proteins. Based on the cis-elements analysis of the *M. sinensis HD-Zip* genes, it appeared that most of the genes were associated with plant growth and development, various hormones, and abiotic stress (Table S3).

### 3.2. Phylogenetic Analysis and Classification of HD-Zip Genes

**Figure 1:**
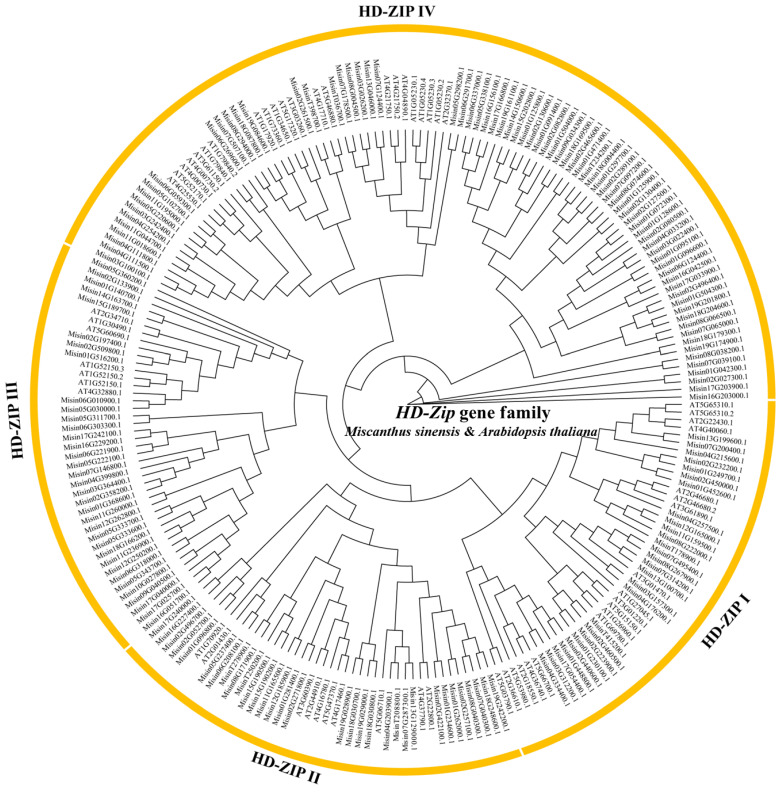
Unrooted phylogenetic tree illustrating links between the 169 HD-Zip proteins in *Miscanthus sinensis* and 47 HD-Zip members in *Arabidopsis thaliana*. The phylogenetic tree was constructed by IQ-TREE.

To explore the classification of HD-Zip protein in *M. sinensis*, the NJ rootless phylogenetic tree was constructed using HD-Zip protein sequences from 169 *M. sinensis* members and 58 *A. thaliana* members. The results showed that the *M. sinensis HD-Zip* gene family was divided into four classes: HD-Zip I–IV. Among the 169 HD-Zip proteins, 27 members were identified from the HD-Zip I subgroup, 32 members were from the HD-Zip II subgroup, 35 members belonged to the HD-Zip III subfamily, and 75 members belonged to the HD-Zip IV subfamily (Figure 1). The HD-Zip I–III subgroups contained a similar number of *M. sinensis* HD-Zip proteins, whereas the HD-Zip IV subgroup contained the largest amount of *M. sinensis* HD-Zip proteins.

### 3.3. Gene Structure and Motif Composition of the HD-Zip Gene Family

**Figure 2:**
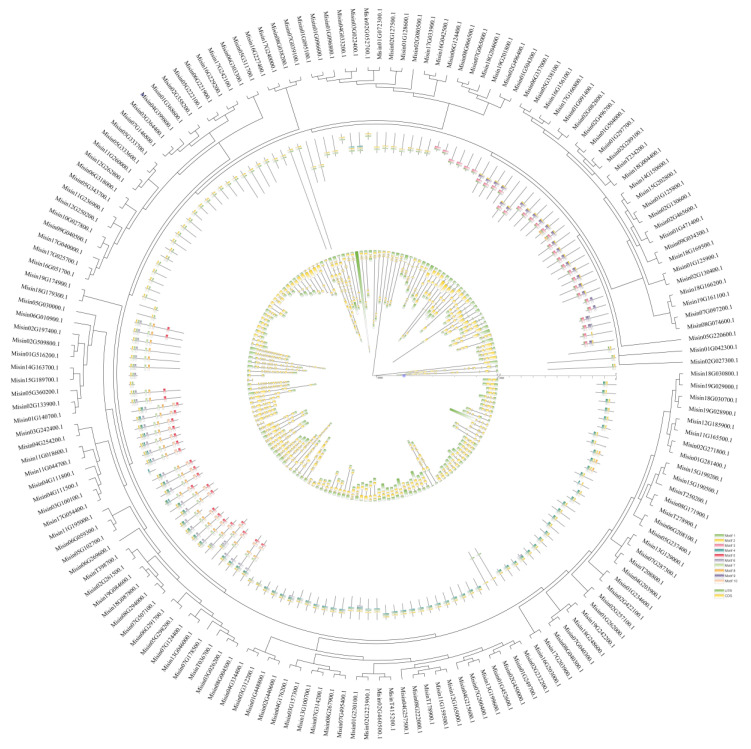
A phylogenetic analysis of *M. sinensis HD-Zip* genes, as well as the pattern of motifs and gene structure. By using MEME, the HD-Zip proteins’ motifs composition was identified, and gray lines indicate non-conserved sequences. An overview of the sequence information for 10 motifs can be found in Figure S1. With the online tool GSDS, the exon-intron structure of *M. sinensis HD-Zip* genes was analyzed. The lengths of exons and introns were proportionately displayed.

In order to further reveal the structural similarity and diversification of *M. sinensis* HD-Zip proteins, the intron and exon structure of *M. sinensis HD-Zip* genes was determined based on ten conserved motifs with the help of a MEME tool (Figure 2). Various lengths of conserved motifs were found, ranging from 19 to 42 amino acids, which showed a highly diverse distribution. Among 169 genes, there were 27 genes with only one intron, whereas the highest number of introns (a total of eighteen) were present in *Misin17G240000.1*, *Misin02G197400.1*, *Misin02G509800.1,* and *Misin01G516200.1*. In addition, the number of exons was a minimum of two and a maximum of eight. Of the proteins, 93.5% had motif 1 and motif 4. Notably, motif 10 was only found in group Ⅳ, which showed that this group may be related to specific functions. The details of the sequence logos of each motif can be found in Figure S1.

### 3.4. Chromosomal Distribution and Gene Duplication of the HD-Zip Genes

**Figure 3:**
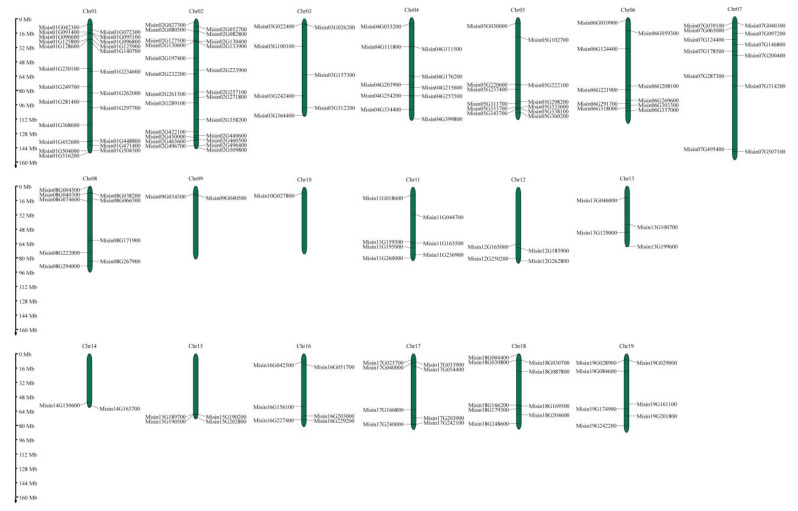
Distribution of 169 *HD-Zip* genes on 19 *M. sinensis* chromosomes, represented by vertical bars, with chromosomes numbered at the top. On the left, the scale represents chromosome length in megabytes (Mb).

A total of 169 *M. sinensis HD-Zip* genes showed an unequal distribution on 19 *M. sinensis* chromosomes (Figure 3). The largest number of *M. sinensis HD-Zip* genes was detected on chromosome 2 (24, ~14.20%), while chromosome 10 contained the least (1, ~0.01%).

**Figure 4:**
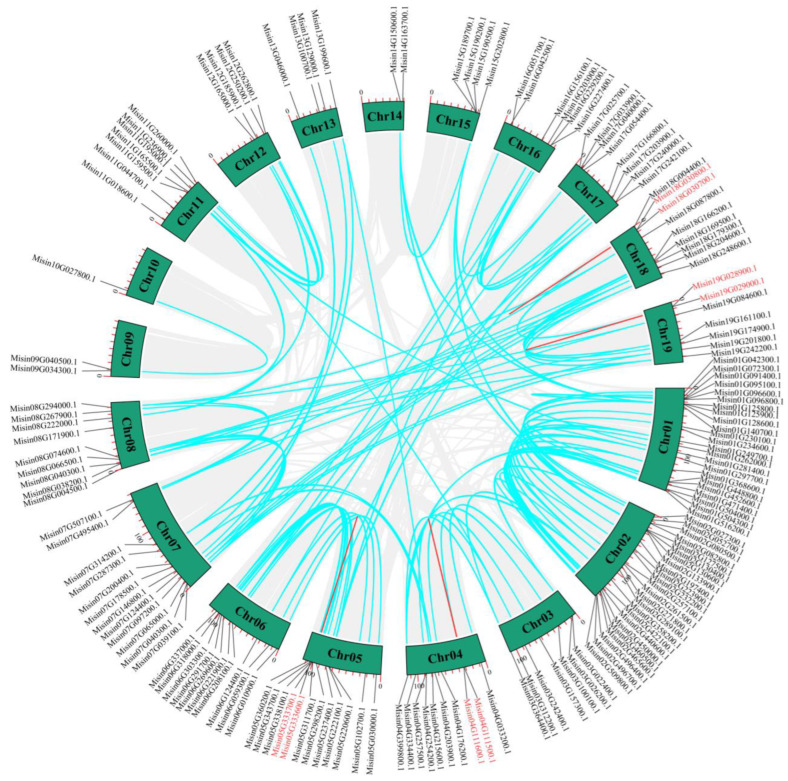
Diagrams depicting the interchromosomal relationship of *M. sinensis HD-Zip* genes. All synteny blocks in the genome were represented by gray lines, while segmental duplicates were shown by blue lines, and tandem duplicates were shown by red lines.

A genome duplication event analysis was carried out for *M. sinensis HD-Zip* genes as well. Tandem and segmental gene duplication could produce a great quantity of gene families. In this study, four pairs of *M. sinensis HD-Zip* genes linked by red lines were examined as tandem repeats (Figure 4), including a pair of tandem repeats on chromosomes 4, 5, 18, and 19, respectively. Also, a total of 72 segmental duplication gene pairs linked by blue lines were identified in the *M. sinensis HD-Zip* gene family (Table S4). According to these findings, some *HD-Zip* genes may have evolved through replication events, which were the main driving forces for the evolution of the *HD-Zip* gene family.

### 3.5. Evolutionary Analysis of HD-Zip Genes in Several Different Species

**Figure 5:**
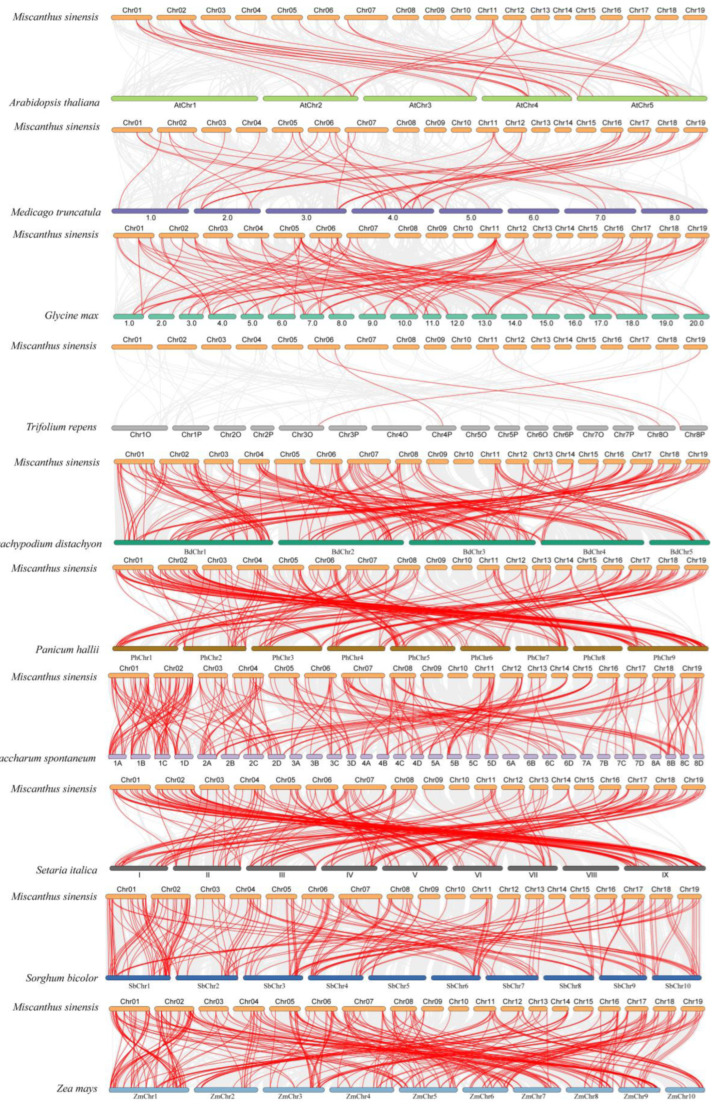
*HD-Zip* gene synteny analysis between *M. sinensis* and ten species. Collinear blocks from *M. sinensis* and other plant genomes were indicated by a gray background line, whereas syntenic *HD-Zip* gene pairs were indicated by a red line.

To deduce the evolutionary relationship of *HD-Zip* genes between *M. sinensis* and related species, comparative syntenic maps were conducted for four dicotyledonous plants (*Arabidopsis thaliana*, *Medicago truncatula*, *Trifolium repens,* and *Glycine max*) and six species of monocotyledons (*Bracypodium distachyon*, *Saccharun spontaneum*, *Panicum hallii*, *Sorghum bicolor*, *Zea mays,* and *Setaria italica*). *M. sinensis* and these ten species showed 28, 43, 4, 95, 187, 242, 195, 202, 268, and 191 homologous pairs, respectively (Table S5). As can be found in Figure 5, there was a large amount of collinearity between *HD-Zip* gene family members of *M. sinensis* and other monocotyledons, which showed that there were differences in the evolutionary branches of dicotyledons and monocotyledons in the process of systematic evolution.

### 3.6. Expression Analysis of HD-Zip Genes during Different Seed Germination Stages of M. sinensis

**Figure 6:**
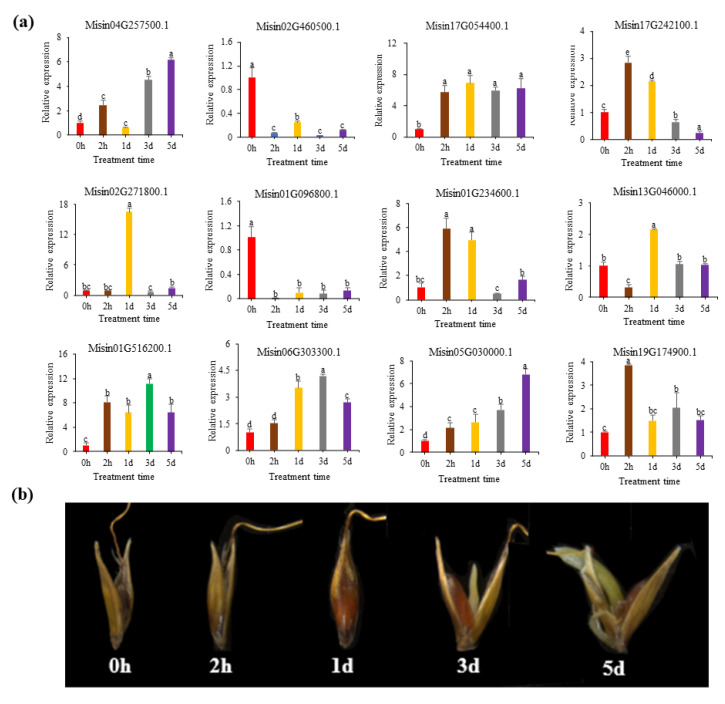
(**a**) Expression patterns of 12 *HD-Zip* genes from *M. sinensis* related to seed germination chosen at random. The gene *Unigene33024* has been used to standardize all the data. Significant differences (*p* < 0.05) between the treatments were denoted by lowercase letter(s) above all the bars. (**b**) Seed morphological changes during seed germination.

A qRT-PCR analysis of *HD-Zip* members was performed to determine their expression during *M. sinensis* seed germination. The expression patterns of 12 randomly selected *HD-Zip* genes during *M. sinensis* seed germination were analyzed (Figure 6). The expression patterns varied in different genes during seed germination, while some genes had similar expression patterns, suggesting that these genes have similar functions. The relative expression levels of *Misin02G460500.1* and *Misin01G096800.1* showed a downward trend, while those of *Misin04G257500.1* and *Misin05G030000.1* were the opposite. Genes whose relative expression level increased first and then decreased were *Misin17G242100.1*, *Misin01G234600.1*, *Misin13G046000.1*, *Misin17G242100.1*, *Misin01G234600.1*, *Misin13G046000.1*, *Misin19G174900.1*, *Misin06G303300.1*, *Misin01G516200.1,* and *Misin02G271800.1*.

Additionally, the expression level of *Misin06G303300.1* was greatly higher than the other 11 genes in the process of seed germination, indicating that *Misin06G303300.1* played a crucial role in seed germination, but the specific function of this gene remains to be studied (Figure S2).

### 3.7. Expression Patterns of HD-Zip Genes in Response to Abiotic Stress

**Figure 7:**
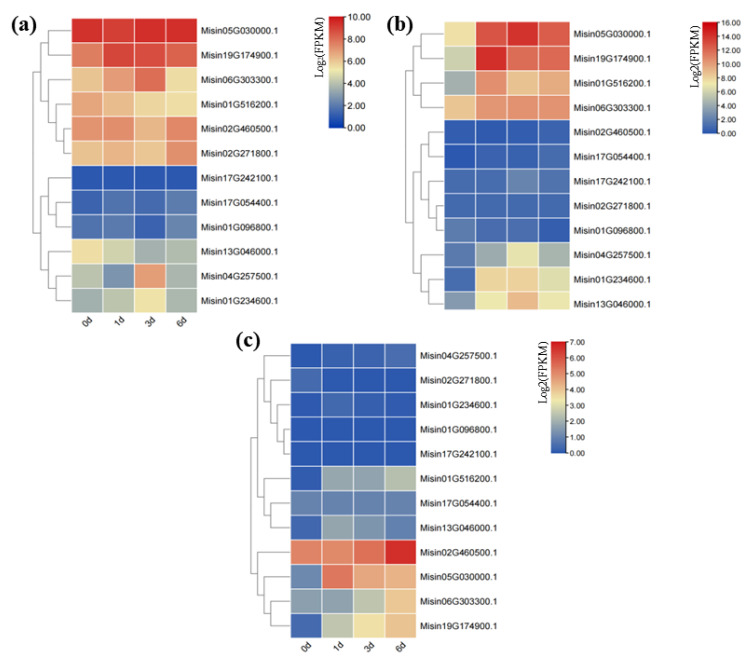
Expression patterns of 12 *M. sinensis HD-Zip* genes in different abiotic stresses: (**a**) a heatmap of 12 *M. sinensis HD-Zip* genes under drought stress; (**b**) a heatmap of 12 *M. sinensis HD-Zip* genes under salt stress; (**c**) a heatmap of 12 *M. sinensis HD-Zip* genes under chromium stress. Expression changes of *M. sinensis HD-Zip* genes under abiotic stresses were shown by log2 change folds, and the levels of expression were illustrated by color gradations.

In order to explore the roles of *HD-Zip* genes involved in responding to osmotic stress, the expression profiles of *HD-Zip* genes in *M. sinensis* root under three stresses were examined. According to the results, the expression levels of these 12 genes under the three stresses were significantly different. The data were presented with clusters using log2 fold-change values (Figure 7). It can be found that the majority of the selected genes were highly expressed in drought stress, and few genes were induced or inhibited by chromium stress and salt stress. Under drought stress, *Misin05G030000.1* and *Misin19G174900.1* were significantly upregulated in *M. sinensis* root. Furthermore, *Misin19G174900.1* was significantly expressed under salt stress and *Misin02G460500.1* was significantly upregulated under chromium stress (log2 based fold change ≥ 6).

## 4. Discussion

As a promising crop for non-food bioenergy, *M. sinensis* is widely planted all over the world and can withstand various abiotic stresses. The *HD-Zip* gene family is one of the most important transcription factors of higher plants, which plays a crucial role in the growth and development of plants, as well as stress responses [18,45]. A recent publication of chromosome scale assembly of the *M. sinensis* genome provided a precious genetic resource for the improvement of *Miscanthus* breeding and the mining of a stress-resistant molecular regulatory network.

Previous studies showed that there is no significant correlation between genome size and *HD-Zip* gene number [46]. Indeed, in this study, there were 169 *HD-Zip* genes in *M. sinensis* (1.88 Gb) [32], 32 members in barley (5.1 Gb) [47,48], 47 members in foxtail millet (423 Mb) [49,50], and 47 members in *Arabidopsis thaliana* (125 Mb) [18,51]. The results were consistent with the previous conclusions. On the other hand, as a dominant factor in species evolution, genome replication events were common in angiosperm evolution, usually resulting in gene family expansion [52]. There were 72 segmental duplication gene pairs and 4 tandem repeats in the *M. sinensis HD-Zip* gene family. It was inferred that gene replication promoted the evolution of the *HD-Zip* gene family in *M. sinensis*. Additionally, *M. sinensis* was a genetic diploid with an allotetraploid history, and the heterotetraploid was thought to originate from 2.5 Mya due to the outbreak of transposon activity [32]. The variation of gene structure was considered to be one of the representative traces of gene family evolution [53]. Different subfamilies had different motif structures and composition, but members in the same branch were similar in domain, as well as in number and length of introns, which indicated that there was a close relationship between the diversity of gene structure and the evolution of the *M. sinensis HD-Zip* gene family. Moreover, *M. sinensis* presented similar intron counts and motif compositions within each HD-Zip subgroup, which further supported phylogenetic grouping.

Genes in the same branch usually have a similar function in a multispecies phylogenetic tree [54]. The potential roles of these HD-Zip proteins could be predicted by clustering their sequences in the phylogenetic tree with those from Arabidopsis. During embryogenesis, *REV*, *PHV,* and *PHB* were responsible for two major processes: establishing bilateral symmetry at the apex and establishing the shoot apical meristem (SAM) [55]. In this study, these three *HD-Zip* genes (*REV*, *PHV,* and *PHB*) were in the same subfamily as *Misin06G303300.1*, indicating that *Misin06G303300.1* may have similar functions to them. Also, it was found that *Misin06G303300.1* was highly expressed in seed germination, and had the highest expression level in early germination, suggesting that this gene was related to the development of mature seed embryos, and then affected seed germination. *ATML1* and *PDF2* are two HD-Zip IV members, the silent expression of which can inhibit epidermal gene expression and seed germination [56]. *Misin19G174900.1,* a gene located in the same subgroup as *ATML1* and *PDF2*, had its relative expression level increased at 2 h before decreasing gradually, showing that the germination rate at 2 h may be higher than that of other time points. In *Arabidopsis*, *ATHB2*, a gene of HD-Zip II subgroup, involved in seed germination acted as a light-induced factor and belonged to the same subfamily as *Misin02G271800.1*, *Misin01G096800.1,* and *Misin01G234600.1*. The result provided us with an opportunity to explore potential roles of these genes in seed germination.

In addition, transcriptional factors typically modulate gene expression in response to stress in plants when the environment stimulates them [57]. In our study, *HD-Zip* genes were actively involved in drought stress, salt stress, and chromium stress, which was consistent with previous studies [58,59,60]. *ATHB13* was a gene belonging to the HD-Zip I subfamily. A study reported that transgenic Arabidopsis overexpressing *JUNGBRUNNEN1* lowered levels of cellular H_2_O_2_ and increased tolerance to drought stress [61]. Transgenic Arabidopsis plants containing *ATHB13*, which acted as an upstream regulator of *JUB1*, were more drought-resistant [62]. During drought conditions, *Misin02G460500.1*, an HD-Zip I gene, was significantly upregulated. Thus, *Misin02G460500.1* might have a similar function to *ATHB13* regarding drought resistance. Members of the HD-Zip III subgroup have been shown to be related to heavy metal, salt, and drought stress. Previous studies have shown that transgenic lines overexpressing miRNA166 inhibited Cd accumulation in rice grains [31]. Also, plants overexpressing miRNA166 showed development changes, such as leaf curl and xylem diameter reduction, so as to improve its drought resistance [63]. There were differences in the expression level between *MtHDZ5*, *MtHDZ13,* and *MtHDZ22*, three *HD-Zip* genes in *Medicago truncatula*, under stress conditions of 180 mM and 200 mM NaCl, indicating their response to salt stress [64]. In our study, *Misin05G030000.1* and *Misin06G303300.1*, two members of HD-Zip class III, were highly expressed under chromium, salt, and drought stress. It can be speculated that *Misin05G030000.1* and *Misin06G303300.1* have similar functions with the above genes in stress resistance.

## 5. Conclusions

In the current study, the *HD-Zip* gene family in *M. sinensis* was first comprehensively and systematically analyzed. One hundred and sixty-nine identified *M. sinensis HD-Zip* members were divided into four subgroups, and their distribution was uneven on 19 chromosomes. In addition, subcellular localization predicted that most of the proteins (~99.41%) were in the nucleus, and all proteins except protein Misin16G051700.1 were transmembrane proteins. According to the cis-elements analysis, it appeared that most of the genes were related to plant growth and development, various hormones, and abiotic stress. Indeed, there were 72 segmental duplication gene pairs and 4 tandem repeats. Gene expression analysis results demonstrated that *Misin06G303300.1* could critically participate in the seed germination of *M. sinensis*. In addition, *Misin05G030000.1* and *Misin06G303300.1* played a potential role responding to chromium, salt, and drought stress. This study will provide a basis of information for future studies of *HD-Zip* genes in stress-responsive traits, as well as possible genetic improvements for *M. sinensis* seed germination.

## Data Availability

Publicly available data sets were analyzed in this study. These data can be found at: https://phytozome-next.jgi.doe.gov/info/Msinensis_v7_1, accessed on 1 June 2022.

## Supplementary Materials

The following supporting information can be downloaded at: https://www.mdpi.com/article/10.3390/genes13122256/s1, Table S1: Primer information for selected *M. sinensis HD-Zip* genes in qRT-PCR validation and reference genes in this study. Table S2: The basic information of all identified *M. sinensis HD-Zip* genes. Table S3: Analysis of cis-acting element of *HD-Zip* genes in *M. sinensis.* Table S4: Tandem and segmental gene duplication of *HD-Zip* genes in *M. sinensis*. Table S5: Homologous pairs between *M. sinensis* and other 10 species. Figure S1: The details of the sequence logos of each motif. Figure S2: Expression profiles of 12 genes at the same time point, including 0 h, 2 h, 1 d, 3 d, 5 d, in response to seed germination. Data were normalized to reference gene *Misin17G054400.1*.
