# Supplementary material for: Genome-Wide Identification and Expression Profile of the HD-Zip Transcription Factor Family Associated with Seed Germination and Abiotic Stress Response in Miscanthus sinensis
Source: Genes (Basel). 2022 Nov 30;13(12):2256. doi: 10.3390/genes13122256 (PMC9777646; doi:10.3390/genes13122256)
Supplement: Supplementary file 1 [file genes-13-02256-s001.zip › Supplementary Figures S1-S2.pdf]

Motif 1

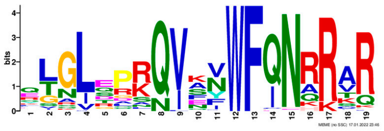

Motif 2

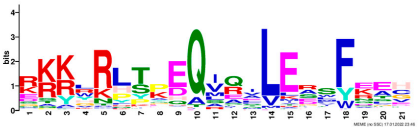

Motif 3

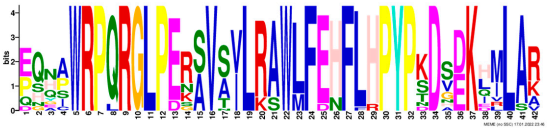

Motif 4

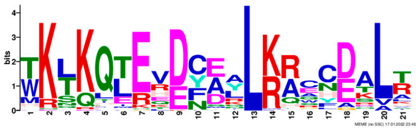

Motif 5

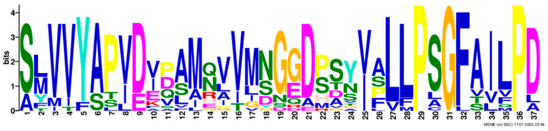

Motif 6

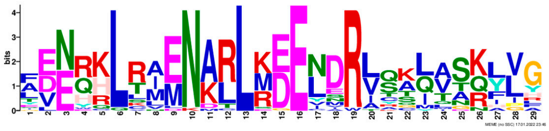

Motif 7

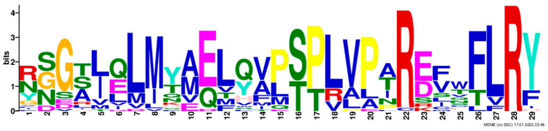

Motif 8

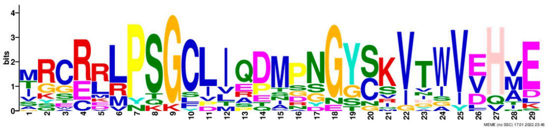

Motif 9

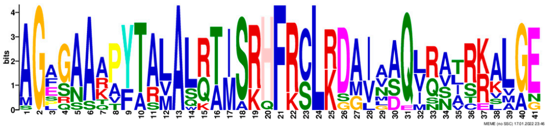

Motif 10

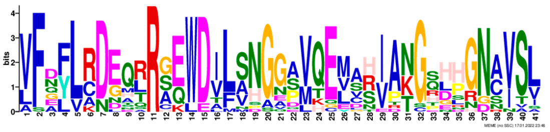

Figure S1. The details of the sequence logos of each motif.

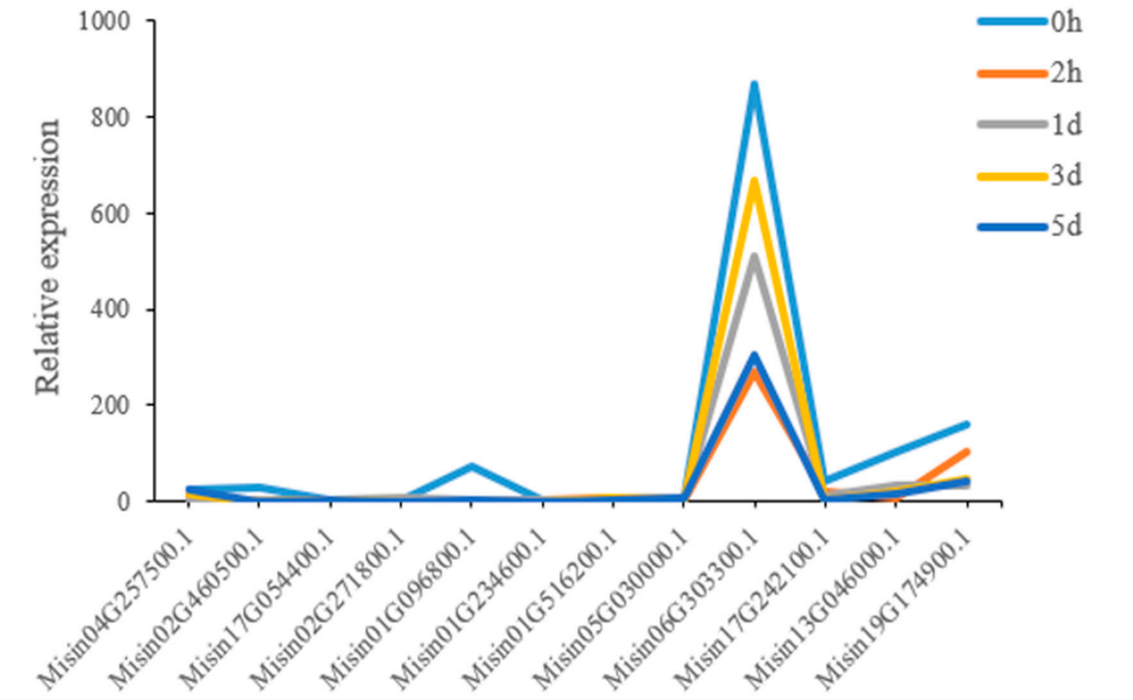

Figure S2. Expression profiles of 12 genes at the same time point, including 0 h, 2 h, 1 d, 3 d, 5 d, in response to seed germination.
